# Assessing ABO/Rh Blood Group Frequency and Association with Asymptomatic Malaria among Blood Donors Attending Arba Minch Blood Bank, South Ethiopia

**DOI:** 10.1155/2016/8043768

**Published:** 2016-01-27

**Authors:** Getaneh Alemu, Mohammedaman Mama

**Affiliations:** Department of Medical Laboratory Science, Arba Minch University, P.O. Box 21, Arba Minch, Ethiopia

## Abstract

*Background*. Determination of the various ABO/Rh blood group distributions and their association with malaria infection has paramount importance in the context of transfusion medicine and malaria control.* Methods*. Facility based cross-sectional study was conducted from February to June, 2015, to assess ABO/Rh blood groups distribution and their association with asymptomatic malaria. A structured questionnaire was used to collect data. Blood grouping was done using monoclonal antibodies. Thin and thick blood films were examined for* Plasmodium* parasites. Data were analyzed using SPSS version 20.0.* Results*. A total of 416 blood donors participated with median age of 22 ± 0.29 (median ± standard error of the mean). Distribution of ABO phenotypes, in decreasing order, was O (175, 42.1%), A (136, 32.7%), B (87, 20.9%), and AB (18, 4.3%). Most of them were Rh+ (386, 92.8%). The overall malaria prevalence was 4.1% (17/416). ABO blood group is significantly associated with malaria infection (*P* = 0.022). High rate of parasitemia was seen in blood group O donors (6.899, *P* = 0.003) compared to those with other ABO blood groups.* Conclusion*. Blood groups O and AB phenotypes are the most and the least ABO blood groups, respectively. There is significant association between ABO blood group and asymptomatic malaria parasitemia.

## 1. Background

The ABO blood group was the first human blood group discovered in 1901 by Landsteiner followed by Rh blood group in 1941 [1, 2]. Currently more than 30 blood groups have been described by the International Society of Blood Transfusion of which only ABO and Rh blood groups remain clinically most important [3, 4]. The ABO blood grouping system consists of the A, B, and H carbohydrate antigens and antibodies against these antigens, while that of Rh is composed of D antigen [5]. Therefore ABO blood grouping is based on the presence or absence of A and B antigens on the surface of red blood cells (RBCs) and Rh grouping is based on the D antigen presence or absence on the RBC surface [6, 7]. The blood group antigens are composed of genetically controlled glycoprotein and glycolipids [7]. All blood group systems are inherited and shared by all human populations but the frequency varies. Those variations depend on the alleles' distribution, mating system of a population, socioeconomic status, ethnic group, and race [8–10].

Since the Second World War, blood and blood component transfusion have been used to correct severe anemia, deficiency of plasma clotting factors, thrombocytopenia, immunodeficiency states, hypoalbuminaemia, and problems related to electrolytes [11, 12]. Transfusion of compatible blood at least for ABO and Rh antigens reduces transfusion reaction in recipients. The ABO and Rh blood groups are also useful in clinical studies, population genetic studies, and researching population migration patterns as well as resolving certain medicolegal issues, particularly of disputed paternity cases [13]. Therefore knowledge of the ABO and Rh blood group distribution in specific population has paramount importance in the context of transfusion medicine. Many previous studies in sub-Saharan Africa reported that blood group O and Rh+ are the most frequent ABO and Rh blood groups, respectively, but the proportion varies by location [5, 14–16].

Ilozumba and Uzozie [17] reported ABO blood group prevalence of 2.63%, 12.05%, 21.05%, and 63.83% for groups AB, B, A, and O, respectively. In another study, Sirina and Clement [16] have reported prevalence of 3.2%, 18.52%, 20.82%, and 57.44% for groups AB, B, A, and O, respectively. Oladeinde et al. [18] also reported that blood groups O (57%) and AB (3.2%) are with the highest and lowest distribution in Benin City of Nigeria. The distribution of ABO blood groups was O (46%), A (27.1%), B (23.1%), and AB (3.8%) according to a study by H. Tadesse and K. Tadesse, in northwest Ethiopia [5]. Similar study in West Gojam, Ethiopia [7], showed frequency distributions of 60%, 26%, and 14% for blood groups of O, A, and B, respectively. Rh+ blood group distribution is much higher than Rh− in Africa according to previous reports [5, 7, 16, 18].

On the other side, both ABO and Rh blood groups have attracted enormous attention regarding their association with genetic and infectious diseases [9]. Previous studies on patients of cancer and tumor [19], heart diseases [20], and parasitic and viral infections [21] indicated associations of ABO and Rh blood groups. In particular, the ABO antigens regulate cellular activities suggesting their impact on determining susceptibility and severity of certain diseases [22]. It has been more than four decades since association of ABO blood group and malaria was suggested. There is also a hypothesis that* Plasmodium falciparum (P. falciparum)* malaria has shaped the distribution of ABO blood groups in humans [23]. Otajevwo [15] and Singh et al. [24] reported significant association between malaria and ABO blood groups where blood group O individuals are more susceptible than other ABO blood groups. However blood group AB individuals were more affected by malaria according to studies by Ilozumba and Uzozie [17] and Oche and Aminu [25]. Regarding disease severity, H. Tadesse and K. Tadesse [5] showed that being of blood group O protects from severe malaria. On the contrary, Sirina and Clement [16], Oladeinde et al. [18], and Epidi et al. [14] indicated that there is no significant association between ABO blood group and malaria parasitemia.

Many other coexisting factors made the study of relationship between malaria and ABO/Rh blood groups difficult that varying findings are reported. Complexity of the interaction between the parasites and host immune responses as well as impact of other RBC polymorphisms may be responsible for such differences [24, 26–30]. Hence the aim of the present study was to assess the ABO/Rh blood group distribution and association with asymptomatic malaria among blood donors attending Arba Minch Blood Bank, South Ethiopia, where malaria is one of the most important public health problems.

## 2. Materials and Methods

### 2.1. Study Design and Area

Facility based cross-sectional study was conducted in Arba Minch Blood Bank from February to June 2015. Arba Minch is located 454 kms south of Addis Ababa, the capital city of Ethiopia. It is found at an altitude of 1200–1300 meters above sea level with an average annual temperature of 29.7°C and rain fall of 900 mm [31]. Arba Minch town and the rural surroundings are one of the most malaria endemic areas of Ethiopia. Arba Minch Blood Bank, located in Arba Minch, is one of the three blood banks found in Southern Nation's, Nationalities, and Peoples Region (SNNPR) of Ethiopia. The blood bank works with an ultimate goal of immediate supply of safe blood for patients who need transfusion and, at average, collects 375 units of blood per month from voluntary donors.

### 2.2. Sample Size and Sampling Technique

All adult population with eligible age range (17–65 years old) for blood donation and living in malaria endemic catchment areas of Arba Minch blood bank were the source population. The study subjects were recruited from those who came to donate blood during the study period. The sample size was determined using single population proportion formula (*n* = [*Z*1 − *α*/2]^2^ *P*(1 − *p*)/*d* ^2^) at 95% confidence level (*Z*  (1 − *ά*/2) = 1.96). A 5% marginal error (*d*) was tolerated and a proportion (*p*) of 0.5 was assumed. Substituting the values, calculated sample size was 384 and the final sample size was 423 after adding 10% to compensate for nonrespondents. Systematic random sampling technique was followed. By tracing back last year's donor flow, we expected 1650 donors during the study period. Therefore, calculated *K* value was 4 (1650/423). The first subject was selected by lottery method and then every fourth voluntary donor has been recruited. The inclusion criterion was passing the clinical screening criteria of the blood bank.

### 2.3. Data Collection

#### 2.3.1. Sociodemographic Data

Nurses who are fluent speakers in the local language were selected and trained for data collection. Sociodemographic data was collected using a pretested structured questionnaire administered through face to face interview. The questionnaire was developed in order to capture data on sociodemographic characteristics, history of malaria, and previous blood donations.

#### 2.3.2. Laboratory Methods

Five milliliters of venous blood was collected from each study participant for ABO/Rh blood grouping and blood film preparation. ABO and Rh blood grouping was done using commercially prepared monoclonal anti-A, anti-B, and anti-D antisera (Agappe Diagnostics Ltd., India) following methods described by Cheesbrough [32]. The rapid forward (cell) grouping method was used. To determine malaria parasitemia, both thin and thick blood films were prepared and examined after staining with 10% Giemsa for 15 minutes. The thin film was fixed with absolute methanol (96% v/v) before staining. For positive samples, asexual stages of malaria parasites were counted against 500 white blood cells (WBC) on the thick film and reported as number of parasites per *μ*L of blood assuming a standard adult leukocyte count of 8000/*μ*L. All laboratory procedures were processed in Arba Minch Blood Bank laboratory by well experienced and trained laboratory technologists following standard procedures.

### 2.4. Statistical Analysis

Data were entered and analysed using statistical package for social sciences (SPSS) version 20.0. Descriptive statistics like frequency, median, and percentage were used to describe the study population characteristics. Bivariate logistic regression was used for assessment of general associations between categorical variables. Multiple logistic regression analysis then followed for variables with *P* ≤ 0.25 in the bivariate analysis. Associations between variables were considered statistically significant only if *P* value ≤0.05 at 95% confidence level.

### 2.5. Data Quality Control

Data collectors were trained on the study procedures. The questionnaire was translated to the local language and retranslated back to English. Investigators supervised all aspects of data collection. Standard operating procedures were strictly followed for blood grouping and malaria diagnosis. Giemsa stalk solution was stored appropriately and the staining quality was checked every week by processing known positive and negative samples. All malaria positive and 10% of negative slides were blindly reexamined by another technologist and all blood groupings were done in duplicate. A maximum of 5% discordant readings were tolerated for malaria diagnosis and a third test (tie-breaker) was conducted during blood grouping in case the duplicate test results are discordant.

### 2.6. Ethical Clearance

Ethical approval for the research was granted by review boards of Arba Minch University College of Medicine and Health Sciences and Arba Minch Blood Bank. Written consent was obtained from all participating blood donors. All laboratory results were communicated to study subjects promptly.

## 3. Results

A total of 416 blood donors participated in the study with response rate of 98.3%. Of the participants, 232 (55.8%) were male and 184 (44.2%) were female. The median age of the study subjects was 22 ± 0.29 (median ± SEM) with a range of 18–59 years. Donors with self-reported malaria history account for 154 (37%). Only 152 (36.5%) donors sleep under bed net. All of the study subjects were voluntary donors and 219 (52.6%) have donated blood before, while the rest 197 (47.4%) came to donate blood for the first time. The most frequent ABO blood group was blood group O, 175 (42.1%), followed by group A accounting for 136 (32.7%) of the study subjects. Blood groups B and AB account for 87 (20.9%) and 18 (4.3%) of the donors, respectively. Most of the donors, 386 (92.8%), were Rh+, while only 30 (7.2%) were Rh− (Table 1). The distribution of various blood groups in relation to sex is summarized in Table 2. Blood groups O and AB are the highest and least distributed ones, respectively, both in males and females. Difference in the occurrence of ABO blood group phenotypes was not statistically significant between male and female donors (*P* = 0.656).

The overall malaria prevalence in the present study was 4.1% (17/416). Eight and 9 donors were infected with* P. falciparum* and* Plasmodium vivax* (*P. vivax*), respectively. Mixed infection was not detected. As can be seen from Table 3, most of malaria infected donors were with low parasitemia (light infection). Higher proportion of females (6.0%, 11/183) were infected than males (2.6%, 6/233) but the difference was not statistically significant (*P* = 0.091).

Bivariate analysis shows that malaria parasitemia was significantly associated with bed net utilization (*P* = 0.047), previous blood donation history (*P* = 0.022), and ABO blood group (*P* = 0.033) but according to logistic regression analysis, only ABO blood group (*P* = 0.022) is associated with malaria parasitemia. High rate of parasitemia was seen in blood group O donors (8%, 14/175) followed by those having blood group AB (5.6%, 1/18). Malaria parasitemia was not significantly associated with Rh blood group (*P* = 0.104) (Table 4). ABO blood group was not significantly associated with both* plasmodium* species (*P* = 0.251) and parasite load (*P* = 0.976).

## 4. Discussion

The malaria prevalence (4.1%) in the present study, which is a reflection of high rate of asymptomatic* plasmodium* parasitemia in endemic areas, seems considerable. Its implication is enormous when viewed from the recipients' side which are already weakened by existing severe diseases. The prevalence would even be much higher if the data was collected during the major malaria transmission season (September to December). Submicroscopic parasitemia is also common in asymptomatic carriers such that use of more sensitive diagnostic tests would yield higher rate of malaria infection. This is evidenced by findings from Ghana that a prevalence of 4.7% by microscopy increased to 18% when diagnosed using polymerase chain reaction [33].

In this study, high percentage of blood group O (42.1%) phenotype was observed among study participants followed by A (32.7%), B (20.9%), and AB (4.3%). This goes in line with some recent studies reporting high group O frequency in malaria rampant tropical regions as compared to group A [5, 7, 14, 16–18]. Studies in malaria free cold regions showed that group A phenotype is more common than O [34, 35]. Hence findings in the present study substantiate the hypothesis that* P. falciparum* has evolutionarily shaped the distribution of ABO phenotype. Other studies in malarious areas of India [36] show that B phenotype is the most abundant ABO blood group suggesting that the above scenario is not exclusive.

We found statistically significant association (*P* = 0.022) between ABO blood group and malaria parasitemia such that donors with blood group O had the predominant rate of infection (8.0%, 14/175). Blood group O donors were 6.899 (95% CI = 1.951–24.391; *P* = 0.003) times more susceptible to* plasmodium* infection than those with other ABO blood groups. This goes in line with findings from previous studies conducted in Nigeria [14, 15]. Contrasting results have been reported in other two studies in Nigeria, where 37.5% and 100% of the cases were with blood group AB [17, 25]. According to studies by Sirina and Clement [16], Otajevwo [15], and Oladeinde et al. [18], ABO blood groups were not significantly associated with malaria infection rate. Complexity of the interaction between the parasites and host immune responses as well as impact of other RBC polymorphisms may be responsible for such differences.

All four ABO phenotypes were infected with* P. falciparum* but only group O donors were parasitemic for* P. vivax.* This agrees with previous reports suggesting that individuals with blood groups A, B, and AB are more susceptible to* P. falciparum* infection than those with O phenotype [37]. Previous reports suggest that non-O-blood groups are associated with complicated or severe malaria because they confer resetting [5, 37]. All infected participants were at the same level of morbidity (asymptomatic) and with comparable parasite load (light infection). Because of this, we could not assess the relation between ABO blood groups and malaria disease severity.

Most of the study participants were Rh+ (92.8%) in this study which goes in line with previous studies [5, 16, 38]. The number of Rh+ participants would be increased if weak-D antigen was detected. Out of 30 Rh− participants, 16 were female that occurrence of hemolytic disease of the new born may be a medical issue in the population. Similarly with previous studies [5, 14, 22, 39], Rh blood group is not associated with asymptomatic malaria infection in the present study.

## 5. Conclusion

Blood groups O and AB phenotypes are the most and the least frequent ABO blood groups, respectively, in the study population. There is considerable prevalence of malaria parasites in apparently healthy blood donors attending Arba Minch Blood Bank. Donors with blood group O are significantly more susceptible to asymptomatic malaria as compared to non-group-O donors. Further studies are recommended to assess the biological basis of association between ABO/Rh blood group and malaria parasitemia.

## Figures and Tables

**Table 1:** Sociodemographic characteristics and clinical history of blood donors attending Arba Minch Blood Bank from February to June 2015.

Variables	Frequency	
	Number (*n*)	Percent (%)
Sex		
Male	232	55.8
Female	184	44.2
Age group		
18–27	355	85.3
28–37	48	11.6
>37	13	3.1
Marital status		
Single	339	81.5
Married	77	18.5
Total	416	100
Educational level		
Illiterate	112	26.9
Primary	21	5.0
Secondary	76	18.3
Tertiary	207	49.8
Occupation		
House wife	14	3.3
Peasant/agriculture	117	28.1
Employed	141	33.9
Business/shop	79	19.1
Student	65	15.6
Previous malaria infection		
Yes	154	37
No	262	63
Time of last malaria episode		
2–6 months	18	11.7
>6 months	84	54.5
Do not remember the time	52	33.8
Use of bed net the previous night		
Yes	152	36.5
No	264	63.5
Previous history of blood donation		
Yes	219	52.6
No	197	47.4
Number of previous donations		
Once	124	56.6
2–4 times	78	35.6
>4 times	17	7.8
ABO blood group		
O	175	42.1
A	136	32.7
B	87	20.9
AB	18	4.3
Rh blood group		
Rh+	386	92.8
Rh−	30	7.2

**Table 2:** Phenotypic distribution of ABO blood group among blood donors attending Arba Minch Blood Bank from February to June 2015.

	ABO blood group				Total
	O	AB	B	A	
Sex					
Male	95 (40.9%)	8 (3.5%)	49 (21.1%)	80 (34.5%)	232 (100%)
Female	80 (43.5%)	10 (5.4%)	38 (20.7%)	56 (30.4%)	184 (100%)

Total	175 (42.1%)	18 (4.3%)	87 (20.9%)	136 (32.7%)	416 (100%)

**Table 3:** Malaria prevalence and mean parasite load among ABO blood group phenotypes of blood donors attending Arba Minch Blood Bank from February to June 2015.

	Examined	Infected		Mean parasite density/*µ*L
		*P. falciparum *	*P. vivax*	
ABO blood group				
O	175	5 (2.6%)	9 (5.1%)	377.14
AB	18	1 (5.6%)	0	320.0
B	87	1 (1.1%)	0	320.0
A	136	1 (0.7%)	0	80.0

Total	416	8 (1.9%)	9 (2.2%)	

**Table 4:** Association of malaria parasitemia with different independent variables among blood donors attending Arba Minch Blood Bank from February to June 2015.

Variable	Number examined	Rate of malaria infection (%)	Crude OR	*P* value	Adjusted OR	*P* value
Sex						
Male	232	6 (2.6%)				
Female	184	11 (6.0%)	2.395 (0.869–6.604)	0.091	2.599 (0.889–7.594)	0.081
Age						
18–27	355	15 (4.2%)				
28–37	48	1 (2.1%)	0.482 (0.062–3.735)	0.640		
>37	13	1 (8.3%)	1.889 (0.230–15.495)			
Educational status						
Illiterate	111	4 (3.6%)	0.73 (0.224–2.382)	0.853		
Primary	21	1 (4.8%)	0.985 (0.12–8.096)			
Secondary	75	2 (2.7%)	0.532 (0.114–2.487)			
Tertiary	209	10 (4.8%)				
Occupation						
House wife	14	1 (7.1%)	5.346 (0.454–63.005)	0.450		
Peasant/agriculture	117	6 (5.1%)	3.757 (0.744–18.976)			
Employed	141	2 (1.4%)				
Business/shop	79	5 (6.3%)	4.696 (0.889–24.794)			
Student	65	3 (4.6%)	3.363 (0.548–20.632)			
Previous malaria infection						
Yes	155	6 (3.9%)	1.081 (0.392–2.984)	0.880		
No	261	11 (4.2%)				
Use of bed net						
Yes	152	1 (0.7%)				
No	264	16 (5.8%)	4.518 (1.019–20.032)	**0.047**	3.990 (0.866–18.384)	0.076
ABO blood type						
A	136	1 (0.7%)	0.126 (0.008–2.107)	**0.033**	0.163 (0.009–2.896)	**0.022**
B	87	1 (1.1%)	0.198 (0.012–3.317)		0.292 (0.016–5.235)	
AB	18	1 (5.6%)				
O	175	14 (8.0%)	1.478 (0.183–11.945)		2.373 (0.266–21.195)	
Rh blood type						
Rh+	386	14 (3.6%)				
Rh−	30	3 (10%)	2.952 (0.799–10.906)	0.104	2.683 (0.624–11.543)	0.185
Previous history of blood donation						
Yes	219	4 (1.8%)				
No	197	13 (6.6%)	3.798 (1.217–11.848)	**0.022**	3.205 (0.973–10.560)	0.056

## Abbreviations

OR: Odds ratio
*P. falciparum*: * Plasmodium falciparum*
*P. vivax*: * Plasmodium vivax*
RBC: Red blood cells
SEM: Standard error of the mean.
